# Patients With Femoral Neck Fractures Are at Risk for Conversion to Arthroplasty After Internal Fixation: A Machine‐learning Algorithm

**DOI:** 10.1097/CORR.0000000000002283

**Published:** 2022-06-21

**Authors:** Anouk van de Kuit, Jacobien H. F. Oosterhoff, Hidde Dijkstra, Sheila Sprague, Sofia Bzovsky, Mohit Bhandari, Marc Swiontkowski, Emil H. Schemitsch, Frank F. A. IJpma, Rudolf W. Poolman, Job N. Doornberg, Laurent A. M. Hendrickx

**Affiliations:** 1 Department of Orthopaedic Surgery, University Medical Center Groningen, University of Groningen, Groningen, the Netherlands; 2 Department of Orthopaedic Surgery, Massachusetts General Hospital, Harvard Medical School, Boston, MA, USA; 3 Department of Orthopaedic Surgery, Amsterdam UMC, University of Amsterdam, Amsterdam, the Netherlands; 4 Department of Trauma Surgery, University Medical Centre Groningen, University of Groningen, Groningen, the Netherlands; 5 Department of Health Research Methods, Evidence and Impact, McMaster University, Hamilton, ON, Canada; 6 Division of Orthopaedic Surgery, Department of Surgery, McMaster University, Hamilton, ON, Canada; 7 Department of Orthopaedic Surgery, University of Minnesota, Minneapolis, MN, USA; 8 Department of Surgery, Western University, London, ON, Canada; 9 Department of Orthopaedic Surgery, University Medical Center Leiden, Leiden University, Leiden, the Netherlands; 10 Department of Orthopaedic and Trauma Surgery, Flinders Medical Centre, Flinders University, Adelaide, Australia; 11 Department of Surgery, Sint-Antonius Ziekenhuis, Nieuwegein, Utrecht, the Netherlands

## Abstract

**Background:**

Femoral neck fractures are common and are frequently treated with internal fixation. A major disadvantage of internal fixation is the substantially high number of conversions to arthroplasty because of nonunion, malunion, avascular necrosis, or implant failure. A clinical prediction model identifying patients at high risk of conversion to arthroplasty may help clinicians in selecting patients who could have benefited from arthroplasty initially.

**Question/purpose:**

What is the predictive performance of a machine‐learning (ML) algorithm to predict conversion to arthroplasty within 24 months after internal fixation in patients with femoral neck fractures?

**Methods:**

We included 875 patients from the Fixation using Alternative Implants for the Treatment of Hip fractures (FAITH) trial. The FAITH trial consisted of patients with low-energy femoral neck fractures who were randomly assigned to receive a sliding hip screw or cancellous screws for internal fixation. Of these patients, 18% (155 of 875) underwent conversion to THA or hemiarthroplasty within the first 24 months. All patients were randomly divided into a training set (80%) and test set (20%). First, we identified 27 potential patient and fracture characteristics that may have been associated with our primary outcome, based on biomechanical rationale and previous studies. Then, random forest algorithms (an ML learning, decision tree–based algorithm that selects variables) identified 10 predictors of conversion: BMI, cardiac disease, Garden classification, use of cardiac medication, use of pulmonary medication, age, lung disease, osteoarthritis, sex, and the level of the fracture line. Based on these variables, five different ML algorithms were trained to identify patterns related to conversion. The predictive performance of these trained ML algorithms was assessed on the training and test sets based on the following performance measures: (1) discrimination (the model’s ability to distinguish patients who had conversion from those who did not; expressed with the area under the receiver operating characteristic curve [AUC]), (2) calibration (the plotted estimated versus the observed probabilities; expressed with the calibration curve intercept and slope), and (3) the overall model performance (Brier score: a composite of discrimination and calibration).

**Results:**

None of the five ML algorithms performed well in predicting conversion to arthroplasty in the training set and the test set; AUCs of the algorithms in the training set ranged from 0.57 to 0.64, slopes of calibration plots ranged from 0.53 to 0.82, calibration intercepts ranged from -0.04 to 0.05, and Brier scores ranged from 0.14 to 0.15. The algorithms were further evaluated in the test set; AUCs ranged from 0.49 to 0.73, calibration slopes ranged from 0.17 to 1.29, calibration intercepts ranged from -1.28 to 0.34, and Brier scores ranged from 0.13 to 0.15.

**Conclusion:**

The predictive performance of the trained algorithms was poor, despite the use of one of the best datasets available worldwide on this subject. If the current dataset consisted of different variables or more patients, the performance may have been better. Also, various reasons for conversion to arthroplasty were pooled in this study, but the separate prediction of underlying pathology (such as, avascular necrosis or nonunion) may be more precise. Finally, it may be possible that it is inherently difficult to predict conversion to arthroplasty based on preoperative variables alone. Therefore, future studies should aim to include more variables and to differentiate between the various reasons for arthroplasty.

**Level of Evidence:**

Level III, prognostic study.

## Introduction

The incidence of hip fractures is increasing worldwide due to an aging society: the global incidence is expected to increase to 2.6 million annually in 2025 and at least 4.5 million cases annually in 2050 [17, 23]. International guidelines suggest that internal fixation is the treatment of choice in minimally displaced (Garden Type 1 or 2) femoral neck fractures [34]. In patients with displaced (Garden Type 3 or 4) fractures, a decision initially must be made about whether to reduce the fracture and internally fix it or to carry out some form of arthroplasty [34]. Furthermore, internal fixation is considered as a treatment in frail, elderly patients because it has a shorter operation time and is associated with less morbidity [2].

A major disadvantage of internal fixation is the high number of reoperations, with a prevalence ranging from 10% to 49% [2]. Causes for reoperation may include mal- or nonunion, avascular necrosis, infection, or impaired function [13, 32]. Failed internal fixation often results in conversion to THA or hemiarthroplasty [26, 31], which is associated with a substantial increase in morbidity, mortality, and costs [55]. Furthermore, arthroplasty after failed fixation (secondary arthroplasty) is associated with worse outcomes compared with primary arthroplasty [4, 14, 26].

Previous studies have demonstrated that patient characteristics (such as female sex, older age, higher BMI), fracture type, and quality of reduction are associated with conversion to arthroplasty after attempted internal fixation [48, 54]. However, it remains challenging for surgeons to translate these risk factors into a patient-specific estimation of the reoperation risk, and this is often performed ad hoc [3, 37]. A prediction model that calculates this patient-specific probability of conversion may help surgeons in selecting patients better suited to primary arthroplasty instead of internal fixation.

Various orthopaedic studies successfully used machine‐learning (ML) algorithms as an alternative approach to developing clinical prediction models [18, 28, 36, 39, 46]. However, the development of these models for femoral neck fracture treatment has not been described.

Therefore, using data from the Fixation using Alternative Implants for the Treatment of Hip fractures (FAITH) trial, a recent international, multicenter, randomized controlled trial that compared a sliding hip screw to cannulated screw fixation, we asked the following: What is the predictive performance of an ML algorithm to predict conversion to arthroplasty within 24 months after internal fixation in patients with femoral neck fractures?

## Patients and Methods

### Guidelines

This study adhered to the Guidelines for Developing and Reporting Machine Learning Predictive Models in Biomedical Research [27] and the Transparent Reporting of Multivariable Prediction Models for Individual Prognosis or Diagnosis (TRIPOD) guidelines [9]. The TRIPOD statement was developed in 2015 and addresses 22 items deemed essential for transparent reporting to identify potential usefulness and risk of prediction models [9].

### Primary Outcome

The primary outcome of this secondary analysis was conversion to THA or hemiarthroplasty within 24 months after internal fixation.

### Patients: FAITH Trial

The FAITH trial consisted of 1079 patients aged 50 years or older, with a low-energy fracture of the femoral neck treated with fracture fixation, suitable for internal fixation. Exclusion criteria were associated major lower extremity injuries, retained hardware around the hip, infection, bone metabolism disorders, and a history of frank dementia. In the trial, patients were randomly assigned to receive a sliding hip screw or cancellous screws between 2008 and 2014 [13]. The trial was performed in 81 clinical sites in the United States, Canada, Australia, the Netherlands, Germany, Norway, the United Kingdom, and India. Patients and surgeons were not blinded to the surgery type, but the data analyst remained blinded to the treatment groups. The primary outcome of the FAITH trial was revision surgery to promote healing, relieve pain, treat infection, or improve function more than 24 months postoperatively, including implant removal before fracture healing, implant exchange to another internal fixation implant or arthroplasty, and soft tissue procedures. The trial protocol and results have been published [12, 13]. Although the total FAITH dataset included 1079 patients, 198 patients without conversion to arthroplasty did not complete 2-year follow-up and were excluded. We performed a competing risk analysis (a survival analysis that incorporates the probability that a patient died before the primary outcome) to evaluate the effects of censored data. We compared the baseline characteristics of patients included and excluded in the final analysis (Supplementary Table 1; http://links.lww.com/CORR/A836). Then, we provided the cause-specific Cox regression model for both conversion to arthroplasty and death (Supplementary Table 2; http://links.lww.com/CORR/A837). Finally, we described the Fine and Gray competing risk regression (Supplementary Table 3; http://links.lww.com/CORR/A838). Also, patients with more than 5% (6 of 1079) missing data were excluded, leaving 875 patients for analysis (Table 1). Of these patients, 51% (446 of 875) had a sliding hip screw and 49% (429 of 875) had cancellous screws for fracture fixation. Among the patients, 61% (531 of 875) were women; the mean age was 71 ± 12 years. Sixty-five percent (571 of 875) of the fractures were nondisplaced (Garden Type I or II), 25% (217 of 875) were classified as Garden Type III, and 10% (87 of 875) were classified as Garden Type IV. Five percent (40 of 875) of patients were admitted from nursing homes, and 17% (146 of 875) of patients depended on walking aids before their femoral neck fracture [13]. Conversion to THA or hemiarthroplasty occurred in 18% (155 of 875) of patients within 24 months. Sixty-five percent (100 of 155) of this group underwent conversion to THA and 35% (55 of 155) underwent conversion to hemiarthroplasty. The most common reasons for conversion to arthroplasty were screw cutout (28% [44 of 155]), avascular necrosis (28% [43 of 155]), nonunion (17% [27 of 155]), and implant loosening (17% [26 of 155]). Other reasons for conversion were infection, implant breakage, pain, and post-traumatic arthrosis (Table 2).

**Table 1.** Patient demographics and fracture characteristics (n = 875 patients)

Patient characteristic	Value
Age in years	71 ± 12
Gender	
Men	39 (344)
Women	61 (531)
Race or ethnicity	
White	82 (719)
African or Caribbean	3 (27)
East Asian	0.9 (8)
South Asian	13 (117)
Hispanic or Latino	0.3 (3)
Native or Aboriginal	0.1 (1)
BMI in kg/m^2^	25 ± 4.5
Prefracture living status	
Institutionalized	5 (40)
Not institutionalized	95 (835)
Use of aid prefracture	17 (146)
Smoking status	
Current smoker	18 (160)
Former smoker	29 (255)
Nonsmoker	53 (460)
Fall from standing	97 (849)
Garden classification	
Garden Type I	49 (430)
Garden Type II	16 (141)
Garden Type III	25 (217)
Garden Type IV	10 (87)
Fracture line	
Basal	7 (63)
Midcervical	31 (274)
Subcapital	61 (538)
Pauwels classification	
Type I	11 (94)
Type II	73 (638)
Type III	16 (143)
Comorbidities	
Cardiac disease	27 (238)
Hypertension	50 (435)
Kidney disease	6 (56)
Lung disease	16 (139)
Cancer	9 (79)
Depression	15 (135)
Dementia	3 (24)
Diabetes mellitus	14 (123)
Osteoarthritis	25 (220)

Data presented as mean ± SD or % (n).

**Table 2.** Reasons for conversion to arthroplasty

Reason for conversion	Number (n = 155)
Screw cutout	28 (44)
Avascular necrosis	28 (43)
Nonunion	17 (27)
Implant loosening	17 (26)
Infection	2 (3)
Implant breakage	2 (3)
Pain	1 (2)
Posttraumatic arthrosis	1 (2)
Other	3 (5)

Data presented as % (n).

### Missing Data

Only 0.28% of the data were missing. Missing data were imputed using the MissForest algorithm [49]. This algorithm imputes missing values in continuous and categorical data, based on averaging regression trees. We chose this algorithm because it outperformed other methods of imputation, especially when complex interactions and nonlinear relations are suspected [49]. This was performed for the variables of BMI (0.57% missing), diabetes treatment (0.23% missing), and injury mechanism (0.11% missing).

### Candidate Input Variables

**Fig. 1:**
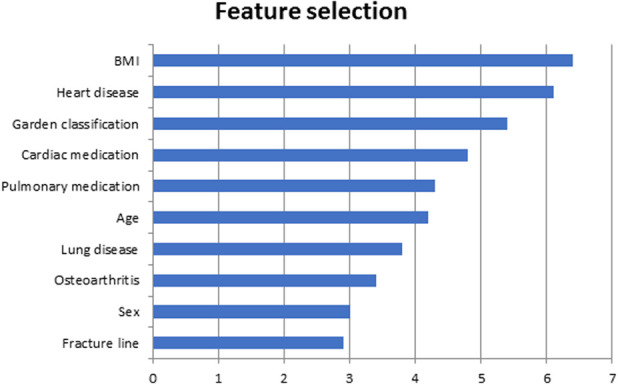
This figure shows variable importance based on feature selection using random forest algorithms.

From the baseline data of the FAITH trial, we identified 27 potential patient and fracture characteristics that may have been associated with our primary outcome, based on biomechanical rationale and previous studies (Table 3) [13, 41, 45, 48, 53]. The randomized treatment (cancellous screws or sliding hip screw) was not included as a separate predictor because we intended to develop a preoperative prediction model, and the FAITH trial showed no advantage for cancellous screws or the sliding hip screw in terms of reoperation [13, 30]. As a first step, variables potentially associated with risk for conversion were identified using random forest algorithms with recursive selection, as previously applied [18, 28, 39]. Random forest is a commonly used technique that works well for various classification and regression tasks. The idea is to first fit a model with all variables and then remove less relevant features [5, 47]. The algorithm identified 10 variables that were relevant to predict the primary outcome (Fig. 1). In order of importance, these variables were: BMI, cardiac disease, Garden classification, use of cardiac medication, use of pulmonary medication, age, lung disease, osteoarthritis, sex, and the level of the fracture line.

**Table 3.** Candidate input variables

Variable	Details
Age	Years
Gender	Men or women
Smoking status	Nonsmoker, previous smoker, current smoker
BMI	kg/m^2^
Prefracture function	Use of aid/nonuse of aid
Prefracture ASA	ASA score 1-5
Race or ethnicity	White, African or Caribbean, East Asian, South Asian, Hispanic or Latino, Native or Aboriginal
Mechanism of injury	Fall from standing or spontaneous
Garden classification	Garden Type I, II, III, and IV
Pauwels classification	Type I, II, and III
Level of fracture line	Subcapital, midcervical, or basal
Additional injuries	Yes or no
Comorbidities: cardiac disease, cancer, hypertension, diabetes, depression, osteoarthritis, lung disease, dementia, kidney disease	Yes or no
Medication use: NSAIDs, cardiac, antihypertensive, diabetes medication, opioids	Yes or no
History of surgery to the hip	Yes or no

### Model Development

The dataset was split into a training set (80%) and test set (20%). The following preexisting Microsoft Azure algorithms were trained to identify patterns related to conversion: Bayes point machine, boosted decision tree, penalized logistic regression algorithm, neural network, and support vector machine [18, 24, 28, 46] (Supplementary Table 4; http://links.lww.com/CORR/A839). These algorithms are supervised forms of ML, meaning that model development relies on the training of the algorithm with labeled data (the presence or absence of conversion). These algorithms were selected because of their successful use in previous studies and their different working mechanisms [10, 19, 25, 35]. The algorithms broadly differ in their method, exploring (non)linearity, average accuracy, and training time. For each algorithm, 10-fold cross-validation was repeated three times in the training set.

### Performance Measures

The predictive performance of the ML models was assessed with the following performance measures: discrimination, calibration, and overall model performance [34]. To assess the discriminative ability of a model, we calculated the area under the receiver operating characteristic (ROC) curve (AUC). The ROC curve plots the sensitivity (true positive rate) against 1 - specificity (false positive rate). The AUC varies from 0.0 to 1.0, and an AUC of 1.0 indicates perfect discriminative ability (which differentiates between patients who had the outcome of conversion to arthroplasty from those who did not) [34]. A prediction model with an AUC above 0.80 was considered as having good discrimination for this study [34].

To assess the calibration of the model, we plotted a calibration curve. The calibration curve is a graphical assessment of the calibration, and it has predictions on the x-axis and the outcome on the y-axis [5, 33]. The calibration curve can be described by the intercept and the slope. The intercept indicates the extent that predictions are systematically too high or too low and should ideally be 0. The slope should ideally be 1. The ideal prediction should therefore be on the 45° line [7]. We consider calibration slopes between 0.90 and 1.10 as sufficient.

To assess the overall model performance, we calculated the Brier score. The Brier score is a composite of discrimination and calibration and is obtained by calculating the squared differences between the actual outcomes and predictions [33, 50]. A Brier score of 0 indicates a perfect model, and a score of 1 is the worst possible. The upper limit of the Brier score is dependent on the incidence of the outcome [50]. After evaluating the performance of the algorithms on the training set, we evaluated the performance of the algorithms on the unseen data of the test set based on the same performance measures.

### Sample Size Justification

The minimum required sample size for this predictive model with a binary outcome, 27 possible predictor parameters, an R^2^ of 0.247 (based on the AUC of 0.858 in Zhu et al. [54]), and an outcome prevalence of 18% is 830 patients [42-44].

### Ethical Approval

The FAITH trial (ClinicalTrials.gov NCT00761813) was approved by the Hamilton Integrated Research Ethics Board (#06-402) and the participating clinical sites’ research ethics boards [13].

### Statistical Analysis

Data preprocessing and analysis was performed using R Version 3.5.2 (The R Foundation), Stata version 15 (StataCorp LP), and Azure (Microsoft Corp). The described algorithms were used in previous studies and are available in Microsoft Azure Machine Learning Studio [18, 28]*.*

## Results

### Performance of ML Prediction Models in the Training Set

**Fig. 2:**
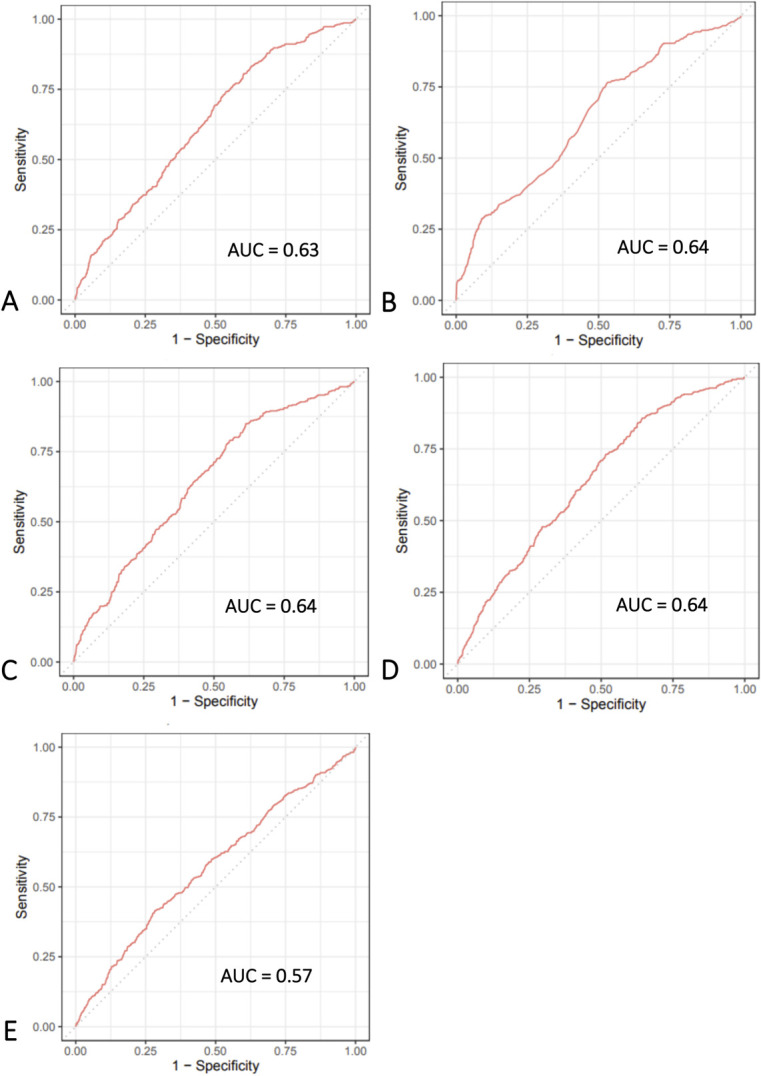
**A-E** These graphs show the receiver operating characteristic curves of the machine-learning models in predicting conversion in the training set for the (**A**) Bayes point machine, (**B**) boosted decision tree, (**C**) penalized logistic regression, (**D**) neural network, and (**E**) support vector machine; AUC = area under the receiver operating characteristic curve.

None of the tested algorithms performed well in the training set (n *=* 700) in predicting which patients would go on to further surgery in the form of arthroplasty: the discriminative performance of the five algorithms, as quantified by the AUC, ranged from 0.57 to 0.64 (Fig. 2). Slopes of the calibration curves ranged from 0.53 to 0.82; intercepts ranged from -0.04 to 0.05. Brier scores ranged from 0.14 to 0.15 (Table 4).

**Table 4.** Performance of ML algorithms in predicting conversion in the training set (n = 700)

Model	AUC	Calibration slope	Calibration intercept	Brier score^a^
Bayes point machine	0.63 (0.60 to 0.66)	0.76 (0.58 to 0.95)	-0.04 (-0.15 to 0.08)	0.14
Boosted decision tree	0.64 (0.61 to 0.67)	0.80 (0.65 to 0.94)	0.00 (-0.12 to 0.12)	0.14
Penalized logistic regression	0.64 (0.61 to 0.67)	0.82 (0.64 to 1.01)	0.00 (-0.12 to 0.12)	0.14
Neural network	0.64 (0.61 to 0.67)	0.53 (0.40 to 0.65)	0.05 (-0.07 to 0.17)	0.14
Support vector machine	0.57 (0.54 to 0.60)	0.54 (0.30 to 0.79)	-0.01 (-0.12 to 0.11)	0.15

a Upper limit of Brier score = 0.15; AUC = area under the receiver operating characteristic curve.

### Performance of ML Prediction Models in the Test Set

In the test set (n *=* 175), AUCs ranged from 0.49 to 0.73. Calibration curve slopes ranged from 0.17 to 1.29 and intercepts ranged from -1.28 to 0.34. Brier scores ranged from 0.13 to 0.15 (Table 5). The upper limit of the Brier score was 0.15, based on an incidence of conversion of 18%. None of the algorithms performed well in the test set in predicting the conversion to arthroplasty.

**Table 5.** Performance of ML algorithms in predicting conversion in the test set (n = 175)

Model	AUC	Calibration slope	Calibration intercept	Brier score^a^
Bayes point machine	0.70	1.09	0.04	0.14
Boosted decision tree	0.64	0.64	-0.54	0.14
Penalized logistic regression	0.73	1.29	0.34	0.13
Neural network	0.67	0.63	-0.34	0.14
Support vector machine	0.49	0.17	-1.28	0.15

a Upper limit of Brier score = 0.15.

## Discussion

This study aimed to develop an ML algorithm to predict conversion to arthroplasty within 24 months after internal fixation of a femoral neck fracture. The ML models were based on patient and fracture characteristics to preoperatively identify patients who were at a high risk of undergoing conversion. However, all ML prediction models showed poor results in predicting the primary outcome, demonstrated by low AUCs and poorly calibrated models.

### Limitations

This study has several limitations. First, the FAITH study is a randomized controlled trial, and therefore includes more homogeneous patients as a result of strict inclusion and exclusion criteria [12, 13]. For example, the FAITH trial excluded patients aged younger than 50 years as well as patients with associated injuries of the lower extremities or soft tissue infections around the hip, cognitive impairment, and disorders of bone metabolism [13]. Developing algorithms in such datasets may result in narrower predictor distributions and are therefore less generalizable to the average population [30].

Second, 198 patients from the FAITH trial were excluded because of incomplete follow‐up. Most (64% [127 of 198]) patients were deceased within 24 months. We compared baseline characteristics with the patients included in the study, showing that the excluded patients were significantly older (p < 0.001), had more comorbidities (p < 0.001), were more often institutionalized (p = 0.008), and dependent on an aid (p < 0.001) (Supplementary Table 1; http://links.lww.com/CORR/A836). Therefore, excluding these patients introduces a substantial bias because we limited our models’ generalizability to those who are healthier. However, we performed a competing risk analysis with the variables included in the final model (Supplementary Table 3; http://links.lww.com/CORR/A838), and showed that hazard ratios for both models were very similar [40]. We recommend that future studies focus on evaluating the feasibility of ML algorithms accounting for competing risk in orthopaedic research [29].

Third, the inclusion of both nondisplaced (Garden I and II) and displaced (Garden III and IV) fractures may skew the results of an ML algorithm. Displaced fractures treated with internal fixation are much more likely to go on to failure compared with nondisplaced fractures [12, 16, 33]. In this study, 35% (304 of 875) of patients had a Garden Types III or IV fracture. In clinical practice, this study is more relevant to patients with Garden Types I and II fractures because they are preferably treated with internal fixation. Ideally, these analyses should be performed in a setting in which we are studying only Garden Types I and II fractures.

None of the tested algorithms performed well in the training set or test set in predicting which patients would subsequently undergo arthroplasty. The conditions for a working ML algorithm were present: We possessed a large dataset from a high-quality randomized controlled trial, and selected variables associated with conversion in previous literature. We propose several reasons as to why the predictive performance was not accurate.

First, despite the demonstrated associations between preoperative characteristics and conversion [16, 48], it is possible that preoperative characteristics alone are insufficient for predicting a multifactorial outcome [54]. Intraoperative and postoperative variables such as the quality of implant positioning, number of screws, and postoperative weightbearing status were not included, but they may have a substantial impact on the patient’s postoperative course and the risk of conversion [54]. Also, preoperative variables of interest that other studies identified as risk factors for conversion were not available in the FAITH database (such as, serum biochemical markers or posterior tilt angle) [54]. Selecting appropriate variables is important in predictive modeling, and in this study, we could not fulfill this criterion completely despite the use of this high‐quality data [34].

Second, the primary outcome of this study was conversion to arthroplasty, which included underlying causes. The most common causes were screw cutout, avascular necrosis, fracture nonunion, and implant loosening. Pathophysiology and risk factors for these underlying mechanisms are partially overlapping but also differ. For example, the Garden classification is an important risk factor for avascular necrosis risk, but it does not necessarily predict the risk of screw cutout or implant loosening [20, 21, 52, 53]. Ideally, subgroup analysis should have been performed to predict these specific outcomes. Despite working with one of the best databases available worldwide, this was not feasible as the underlying pathology was splintered into small subgroups.

Third, we used five ML algorithms to predict the risk of conversion. Although ML-derived prediction models have great potential in risk stratification [37], a recent study showed that the regression-derived probability estimates seem comparable between ML algorithms and logistic regression for binary events in musculoskeletal trauma studies [38]. However, the penalized logistic regression was also used in this study, which is comparable to a logistic regression model. Also, as previous studies successfully used ML algorithms in predicting their orthopaedic outcomes accurately [18, 28, 39], they seem to work. However, ML algorithms cannot perform miracles, and human contributions are pivotal to maximize the predictive performance. Even state-of-the-art ML algorithms, as presented in this paper, cannot leverage information that is simply not present in the data [8, 38, 51].

### Future Perspectives

In the future, prospective studies should include more relevant variables as described in previous literature, such as the quality of reduction, serum biomarkers, and the posterior tilt angle [54]. Furthermore, to understand the pathophysiology for conversion to arthroplasty, large, prospective studies focused on the prediction of underlying etiology are necessary. Also, other artificial intelligence applications could be useful in preoperatively assessing the risk of conversion. Convolutional neural networks (an unsupervised branch of artificial intelligence that is often used to analyze images) may be a valuable adjunct to analyzing pelvic radiographs in identifying fracture characteristics more accurately [1]. Also, three-dimensional CT scans may provide exact information about femoral head displacement, and quantifying this issue may help us predict avascular necrosis risk [11]. Finally, advances in ML techniques, in general, may also aid in developing more accurate prediction models in the future.

### Conclusion

We aimed to develop an ML model that could predict conversion to arthroplasty after internal fixation in femoral neck fractures based on preoperative characteristics. The predictive performance of the trained algorithms was poor, despite the use of one of the best datasets available worldwide. It may be possible that if the current dataset consisted of different variables or more patients, the performance may have been better. Also, various reasons for conversion to arthroplasty were pooled in this study, but predicting underlying etiology may be more precise. Finally, it may be possible that it is inherently difficult to predict conversion to arthroplasty based on preoperative variables alone. Therefore, future studies should aim to include more variables and to differentiate between the different reasons for arthroplasty.

## Group Authors

Members of the Machine Learning Consortium include: Mohit Bhandari, Anne Eva J. Bulstra, Sofia Bzovsky, Job N. Doornberg, J. Carel Goslings, Laurent A.M. Hendrickx, Ruurd L. Jaarsma, Kyle J. Jeray, Gino M.M.J. Kerkhoffs, Jacobien H.F. Oosterhoff, Brad Petrisor, David Ring, Emil H. Schemitsch, Marc Swiontkowski, David Sanders, Sheila Sprague, Paul Tornetta III, and Stephen D. Walter.

Members of the FAITH study include: Writing Committee: Mohit Bhandari, PJ Devereaux, Gordon Guyatt, Lehana Thabane, Stephen D. Walter, Martin J. Heetveld, Kyle J. Jeray, Susan Liew, Emil H. Schemitsch, Paul Tornetta III, Gregory J. Della Rocca, Richard E. Buckley, Robert McCormack, Todd M. Oliver, Michiel J.M. Segers, Amar Rangan, Martin Richardson, Sheila Sprague, Gerard P. Slobogean, Taryn Scott, Julie Agel, Alisha Garibaldi, Qi Zhou, Diane Heels-Ansdell, Helena Viveiros, Stephanie M. Zielinski, Esther M.M. Van Lieshout, Herman Johal, Birgit C. Hanusch, and Marc Swiontkowski.

Steering Committee: Mohit Bhandari (Chair), Marc Swiontkowski, PJ Devereaux, Gordon Guyatt, Martin J. Heetveld, Kyle Jeray, Susan Liew, Martin Richardson, Emil H. Schemitsch, Lehana Thabane, Paul Tornetta III, and Stephen D. Walter.

Global Methods Centre: Mohit Bhandari (Principal Investigator); Sheila Sprague (Research Methodologist); Paula McKay (Manager); Taryn Scott, Alisha Garibaldi, Helena Viveiros, Marilyn Swinton (Research Coordination); Mark Gichuru, Sofia Bzovsky (Adjudication Coordination); Diane Heels-Ansdell, Qi Zhou (Statistical Analysis); Lisa Buckingham, Aravin Duraikannan (Data Management); Deborah Maddock, Nicole Simunovic (Grants Management).

United States Methods Center: Marc Swiontkowski (Principal Investigator); Julie Agel (Research Coordination).

Netherlands Method Center: Martin J. Heetveld (Principal Investigator); Esther M.M. Van Lieshout (Research Coordination); Stephanie M. Zielinski (Trial Coordination).

United Kingdom Method Center: Amar Rangan (Principal Investigator); Birgit C. Hanusch, Lucksy Kottam, Rachel Clarkson (Research Coordination).

Adjudication Committee: Gregory J. Della Rocca (Chair), Robert Haverlag, Susan Liew, Gerard P. Slobogean, Kyle Jeray.
